# “To Be Microbiocidal and Not to Be Cytotoxic at the Same Time…”—Silver Nanoparticles and Their Main Role on the Surface of Titanium Alloy Implants

**DOI:** 10.3390/jcm8030334

**Published:** 2019-03-10

**Authors:** Aleksandra Radtke, Marlena Grodzicka, Michalina Ehlert, Tomasz Jędrzejewski, Magdalena Wypij, Patrycja Golińska

**Affiliations:** 1 Faculty of Chemistry, Nicolaus Copernicus University in Toruń, Gagarina 7, 87-100 Toruń, Poland; Marlena.Grodzicka@doktorant.umk.pl (M.G.); m.ehlert@doktorant.umk.pl (M.E.); 2 Nano-implant Ltd., Gagarina 5/102, 87-100 Toruń, Poland; 3 Faculty of Biology and Environmental Protection, Nicolaus Copernicus University in Toruń, Lwowska 1, 87-100 Toruń, Poland; tomaszj@umk.pl (T.J.); mwypij@umk.pl (M.W.); golinska@umk.pl (P.G.)

**Keywords:** silver nanoparticles, titanium alloy, titanium dioxide nanotubes, silver ions release, biointegration, antimicrobial activity

## Abstract

The chemical vapor deposition (CVD) method has been used to produce dispersed silver nanoparticles (AgNPs) on the surface of titanium alloy (Ti6Al4V) and nanotubular modified titanium alloys (Ti6Al4V/TNT5), leading to the formation of Ti6Al4V/AgNPs and Ti6Al4V/TNT5/AgNPs systems with different contents of metallic silver particles. Their surface morphology and silver particles arrangement were characterized by scanning electron microscopy (SEM), energy dispersive X-ray spectrometry (EDS), and atomic force microscopy (AFM). The wettability and surface free energy of these materials were investigated on the basis of contact angle measurements. The degree of silver ion release from the surface of the studied systems immersed in phosphate buffered saline solution (PBS) was estimated using inductively coupled plasma ionization mass spectrometry (ICP-MS). The biocompatibility of the analyzed materials was estimated based on the fibroblasts and osteoblasts adhesion and proliferation, while their microbiocidal properties were determined against Gram-positive and Gram-negative bacteria, and yeasts. The results of our works proved the high antimicrobial activity and biocompatibility of all the studied systems. Among them, Ti6Al4V/TNT5/0.6AgNPs contained the lowest amount of AgNPs, but still revealed optimal biointegration properties and high biocidal properties. This is the biomaterial that possesses the desired biological properties, in which the potential toxicity is minimized by minimizing the number of silver nanoparticles.

## 1. Introduction

Silver as an antibacterial agent was known and applied in antiquity [1,2,3], but its wide use in different fields of our contemporary life is a result of more and more detailed studies on the mechanisms of Ag antimicrobial activity [4,5,6]. Moreover, the development of modern technologies, which allow for the production of silver nanoparticles (AgNPs) of different sizes, shapes, and properties, is of great importance [7,8,9]. The bactericidal and fungicidal activity of AgNPs has not been fully explained yet, but the three most probable mechanisms are proposed [10,11,12,13]. The first of them assumes the capture of free silver ions, which interferes with ATP production and DNA replication. The second one assumes the generation of reactive oxygen species (ROS) by nanoparticles and silver ions. The produced ROS may also have an adverse effect on DNA, the cell membrane and the membrane proteins [14]. The third mechanism takes into consideration the damage of the bacteria cell membrane as a result of its direct contact with AgNPs. In this case, silver nanoparticles joining the proteins of the cell membrane through the connection with sulfur cause a change in its structure [13,15,16,17,18]. Extensive use of silver nanoparticles, especially in medicine, forces us to think about the potential toxicity of silver to the human body. In some cases, nanoparticles may be toxic for human organisms and the prolonged use of specimens containing silver may cause argyria [19,20]. The potential risk of AgNPs lies in their ability to bioaccumulation in the body [21]. The harmful effects of nanosilver on human cells act according to similar mechanisms, which was mentioned earlier for bacteria. AgNPs accumulate outside the mitochondria, leading to ROS production, which causes mitochondrial damage and interruption of ATP synthesis. Moreover, the interaction of nanosilver with DNA leads to cell cycle stopping [22,23,24,25]. The analysis of previous reports exhibited that the potential toxicity of silver nanoparticles to the human body depends on their size and shape. According to these data, nanoparticles of a diameter smaller than 25 nm and a silver concentration higher than 60 mg/L are considered to be cytotoxic to mammalian cells. Simultaneously, it should be noted that a harmful effect of ionic silver on eukaryotic organisms was noticed at a concentration of 1 mg/L [26,27]. On the other hand, the increase of the AgNPs diameter above 25 nm caused these nanoparticles to be toxic mainly to microorganisms and not harmful to the human body [28,29]. Particles of such sizes are used in different technologies, in which the necessity of a bacteria-free environment exists. One of the interesting directions in the using of AgNPs is combining them with other materials, e.g., polymeric or ceramic ones, in order to form nanocomposites of unique physicochemical properties and bioactivity [30,31,32]. This approach is developed by us in the design and fabrication of new generation surgical titanium/titanium alloy implants, whose surface is enriched with AgNPs as the anti-inflammatory factor [33,34]. To avoid the inflammation effect after implantation, we enriched the titanium alloy implants’ surface with dispersed silver nanoparticles of appropriate diameters using the chemical vapor deposition (CVD) or atomic layer deposition (ALD) techniques. The monitoring of the influence of silver nanoparticles on the adhesion and proliferation of fibroblasts on the implant surface revealed the very diverse biointegration properties of AgNPs-enriched surfaces [35,36]. It should be noted that in our work, two types of Ti6Al4V implants surface were studied, i.e., pure metallic implants and implants with a surface modified by the titania nanotube coating. Analysis of the results of our earlier works revealed that the different surface properties of the tested implants influence the different AgNPs deposition courses and, thereby, their different bioactivities. 

Therefore, determining the direct impact of the deposited silver nanoparticles amount, the form in which they act and their size on their biointegration properties and antimicrobial activity is an important issue. Such an analysis is crucial for the production of implants with a surface, which will be microbiocidal enough but at the same time, optimally biointegral. For this purpose, the surfaces of Ti6Al4V and Ti6Al4V/TNT substrates were coated with different amounts of silver using the CVD technique. This technique enables for the deposition of pure silver nanoparticles on the surface of substrates with complex shapes [37]. We set ourselves the goal of enriching the surface of the titanium alloy implant (non-modified and nanotubular modified) with the smallest number of silver nanoparticles, which will have high antimicrobial properties, and which will not interfere with the adhesion and proliferation of fibroblasts and osteoblasts. We wanted, in this way, to create a biomaterial with the desired bioactivity (microbiocidal and biocompatible), minimizing the number of silver precursor used in the CVD process, thereby minimizing the number of silver nanoparticles on the surface of the layer and minimizing the potential silver toxicity. The results of our works are discussed in this paper.

## 2. Materials and Methods

### 2.1. Substrates

In all our experiments, the Ti6Al4V foil (grade 5, 99.7% purity, 0.20 mm thick (Strem Chemicals, Inc., Bischheim, France), 7 mm × 7 mm pieces) was used as a substrate. Before the anodization process, the Ti6Al4V foil samples were polished with sandpaper; cleaned by ultrasonication for 15 min in acetone, ethanol, and distilled water; and dried in an argon stream. Then the samples were chemically etched in a 1:4:5 mixture of HF:HNO_3_:H_2_O for 30 s, rinsed in deionized water and dried in an argon stream. The Ti6Al4V/TNT5 systems were produced using the electrochemical oxidation method in accordance with the previously described procedures [38]. The uniform TNT5 coatings were produced on the surface of Ti6Al4V substrates using a potential of U = 5 V at room temperature (RT) and at an anodization time t = 20 min. After anodization, all the produced Ti6Al4V/TNT5 samples were washed in deionized water (10 min in an ultrasonic bath) and then their surfaces were drying in a stream of argon at RT and additionally dried at 123 °C. The morphology of the produced coatings was studied using a Quanta scanning electron microscope with field emission (SEM, Quanta 3D FEG, Huston, TX, USA).

### 2.2. Chemical Vapor Deposition of Silver Nanoparticles (AgNPs)

The Ti6Al4V and Ti6Al4V/TNT5 samples were enriched with the AgNPs using the CVD technique, under conditions given in Table 1. Ag_5_(O_2_CC_2_F_5_)_5_(H_2_O)_3_ has been used as a metallic silver CVD precursor. Its synthesis and physicochemical properties were earlier described [36]. The morphology of created coatings was visualized using a scanning electron microscope (Quanta 3D FEG, Huston, TX, USA). The density of the AgNPs aggregation was illustrated using energy-dispersive X-ray spectroscopy (Quantax 200 XFlash 4010, Bruker AXS, Karlsruhe, Germany)). The surface topography was examined by means of atomic force microscopy (AFM, Veeco Metrology Group (Digital Instruments, Santa Barbara, CA, USA) cooperated with NanoScope IIIa controller and MultiMode microscope) using a contactless module with a force of 20 nN in the 2 × 2 μm area.

### 2.3. The Wettability and Surface Free Energy of Biomaterials

In order to evaluate how well (or how poorly) the liquid spreads on the surface of the tested biomaterials, the seated drop method was applied. The contact angle was determined using a goniometer with a drop shape analysis software (DSA 10 Krüss GmbH, Hamburg, Germany). Two liquids—distilled water (H_2_O) and diiodomethane (CH_2_I_2_)—were the reagents chosen to measure the contact angle. For distilled water, the volume of the drop in the contact angle measurement was 3 μL, and in the case of diiodomethane, 4 μL. The measurement was carried out immediately after the drops were deposited. In order to determine the surface free energy (SFE), mathematical calculations were made using the Owens–Wendt model [39]. The measurement with both liquids was performed three times for all tested samples.

### 2.4. Silver Ion Release in the Body Fluid Environment

The studies of silver ions release from the surface of Ti6Al4V/AgNPs and Ti6Al4V/TNT5/AgNPs samples were carried according to the previously used procedure [36]. The pieces of 7 mm × 7 mm samples were immersed in 15 mL of phosphate buffered saline (PBS) in a sealed bottle at a temperature 37 °C for 1, 2, 3, 7, 10, 14, 21, 28 and 34 days. Released concentrations of silver ions in phosphate-buffered saline (PBS) were measured by mass spectrometry with plasma ionization inductively coupled to a quadrupole analyzer using an ICP-MS 7500 CX spectrometer with an Agilent Technologies collision chamber (Agilent Technologies Inc., Tokyo, Japan).

### 2.5. Biointegration Studies

#### 2.5.1. Cell Culture

Murine fibroblast cell line L929 (American Type Culture Collection, Manassas, VA, USA) was cultured at 37 °C in 5% CO_2_ and 95% humidity in a complete RPMI 1640 medium containing 2 mM L-glutamine, heat-inactivated 10% fetal bovine serum (FBS) and antibiotics (100 µg/mL streptomycin and 100 IU/mL penicillin) (all compounds was from Sigma-Aldrich, Darmstadt, Germany). L929 cells were grown in 25 cm^2^ cell culture flasks and the culture medium was changed every 2–3 days. The cells were passaged using a cell scraper when reaching 70–80% confluency.

Human osteoblast-like MG 63 cells (European Collection of Cell Cultures, Salisbury, UK) were plated in a 25 cm^2^ cell culture flask and cultured with Eagle’s Minimum Essential Medium containing 2 mM L-glutamine, 1 mM sodium pyruvate, MEM non-essential amino acid, heat-inactivated 10% FBS and antibiotics (100 µg/mL streptomycin and 100 IU/mL penicillin) (all reagents were purchased from Sigma-Aldrich). The cells were grown at 37 °C in an incubator providing a humidified (95%) atmosphere containing 5% of CO_2_. The culture medium was changed every 2–3 days. The cells were passaged using a 0.25% trypsin-EDTA solution (Sigma-Aldrich) when reaching 70–80% of confluency.

#### 2.5.2. Cell Adhesion and Proliferation Detected by the MTT Assay

L929 fibroblasts, as well as MG-63 osteoblasts, in a volume of 1 mL of appropriate complete culture medium were seeded onto the autoclaved tested nanolayers placed in a 24-well culture plate (Corning, NY, USA) at a density of 1 × 10^4^ cells/well for 24, 72 or 120 h, respectively. The cell adhesion (measured after 24 h) and proliferation (evaluated after 72 h and 120 h) was studied by the MTT (3-(4,5-dimethylthiazole-2-yl)-2,5-diphenyl tetrazolium bromide; Sigma Aldrich) assay using the same method as it was reported in Reference [33]. Briefly, after the respective incubation time, the plates were transferred to a new 24-well culture plate. The MTT solution (5 mg/mL; Sigma-Aldrich) in an appropriate culture medium without phenol red (RPMI 1640 medium for L929 fibroblasts or Eagle’s Minimum Essential Medium for MG-63 osteoblasts; both from Sigma-Aldrich) was added to each well. After 3 h of incubation, the solution was aspirated and 500 μL of dimethyl sulfoxide (DMSO; 100% *v*/*v*; Sigma Aldrich) was added to each well. Finally, the plates were shaken for 10 min and the absorbance was measured at a wavelength of 570 nm with a subtraction of 630 nm (background) using a microplate reader (Synergy HT; BioTek, Winooski, VT, USA). All measurements were done in duplicate in five independent experiments.

#### 2.5.3. Cell Morphology Evaluated by Scanning Electron Microscopy

L929 fibroblasts and MG-63 osteoblasts (1 × 10^4^ cells/well) were incubated on the tested specimens for 24, 72 and 120 h, respectively. Scanning electron microscopy (SEM; Quanta 3D FEG; Carl Zeiss, Göttingen, Germany) analyses were performed to study the morphology changes of the cells grown on the surface of tested plates using the same method as in Reference [33]. Briefly, after the selected incubation time, the samples were fixed in a 2.5% *v*/*v* glutaraldehyde (Sigma Aldrich) and dehydrated in a graded series of ethanol (50%, 75%, 90%, and 100%). Finally, the specimens were dried in vacuum-assisted desiccators overnight and stored at room temperature until the SEM analysis was performed.

#### 2.5.4. Statistical Analysis in the MTT Assay

All values are reported as means ± standard error of the means (SEM) and were analyzed using the nonparametric Kruskal–Wallis one-way ANOVA test with the level of significance set at *p* < 0.05. Statistical analyses were performed with GraphPad Prism 7.0 (La Jolla, CA, USA).

### 2.6. The Evaluation of the Antibacterial Properties of the Ti6Al4V/AgNPs and Ti6Al4V/TNT5/AgNPs Samples

The antimicrobial activity of titanium alloys, Ti6Al4V and Ti6Al4V/TNT5, coated with silver nanograins was studied against Gram-positive (*Staphylococcus aureus* ATCC 6538 and *S. aureus* ATCC 25923=PCM 2054) and Gram-negative (*Escherichia coli* ATCC 8739 and *E. coli* ATCC 25922=PCM 2057) bacteria and yeasts of *Candida albicans* ATCC 10231. Sterile sample plates (7 × 7 mm pieces, 0.2 mm thick) were placed in 1 mL of phosphate buffered saline (PBS) without ions (EURx) for 24 h, and 14 and 28 days to allow for the silver ions to be released. PBS was sterilized with cellulose filters (ø 0.2 µm) prior to use. Ti6Al4V/AgNPs and Ti6Al4V/TNT5/AgNPs plates were then removed and PBS was inoculated with the tested microorganism (final concentration of microorganism in each sample was approximately 5 × 10^5^ c.f.u mL^−1^). Microbial inoculum density was estimated by colony counts. Briefly, the microbial inoculum (approximately 5 × 10^5^ c.f.u. mL^−1^) in sterile PBS was diluted (1:1000) and 100 μL was then spread over the surface of Trypticase Soy Agar (TSA, Becton Dickinson, Franklin lake, NJ, USA) or Sabouraud Dextrose Agar (SDA, Becton Dickinson, Franklin lake, NJ, USA). After incubation, the presence of approximately 50 colonies indicated an inoculum density of 5 × 10^5^ c.f.u. mL^−1^.

Inoculated samples were incubated at 37 °C for 24 h. Subsequently, serial ten-fold dilutions of each sample were prepared. Aliquots (100 µL) of each dilution was spread over the surface of Trypticase Soy Agar (TSA, Becton Dickinson, Franklin lake, NJ, USA) or Sabouraud Dextrose Agar (SDA, Becton Dickinson, Franklin lake, NJ, USA) plates, which had been dried for 15 min prior to inoculation. TSA and SDA media were used for bacterial and fungal growth, respectively. The positive control was Ti6Al4V or Ti6Al4V/TNT5 plates non-coated with AgNPs. Tests were performed in triplicate. Colony forming units were counted on the inoculated plates and compared with the appropriate control plates to estimate the reduction of bacterial or fungal growth. 

The antibacterial rate was calculated using the following formula:

*R* = ((*B* − *A*)/*B*) × 100%,

where *R* is the antimicrobial rate (%), *B* is the average number of microorganisms in PBS after the use of uncovered titanium alloys, and *A* is the average number of microorganisms in PBS after the use of titanium alloys enriched with silver nanoparticles.

## 3. Results

### 3.1. The Fabrication of Ti6Al4V/AgNPs and Ti6Al4V/TNT5/AgNPs Systems

Silver nanoparticles were deposited on the surface of Ti6Al4V and Ti6Al4V/TNT5 substrates using the CVD method (*hot wall* reactor, precursor: Ag_5_(O_2_CC_2_F_5_)_5_(H_2_O)_3_) in the conditions listed in Table 1. The use of the following CVD precursor masses, *m* = 5, 10, 20, 50 mg, made it possible to produce coatings of the AgNPs content: c.a. 0.9, 1.1, 1.3, 2.3 wt% on the surface of Ti6Al4V substrates and 0.6, 1.0, 1.6, 2.3 wt% on the surface of Ti6Al4V/TNT5, respectively (wt% of AgNPs was determined based on the mass sample difference before and after CVD process). Considering the wt% of deposited silver, the studied specimens were marked as Ti6Al4V/0.9–2.3AgNPs and Ti6Al4V/TNT5/0.6–2.3AgNPs.

### 3.2. Surface Morphology and Topography

**Figure 1:**
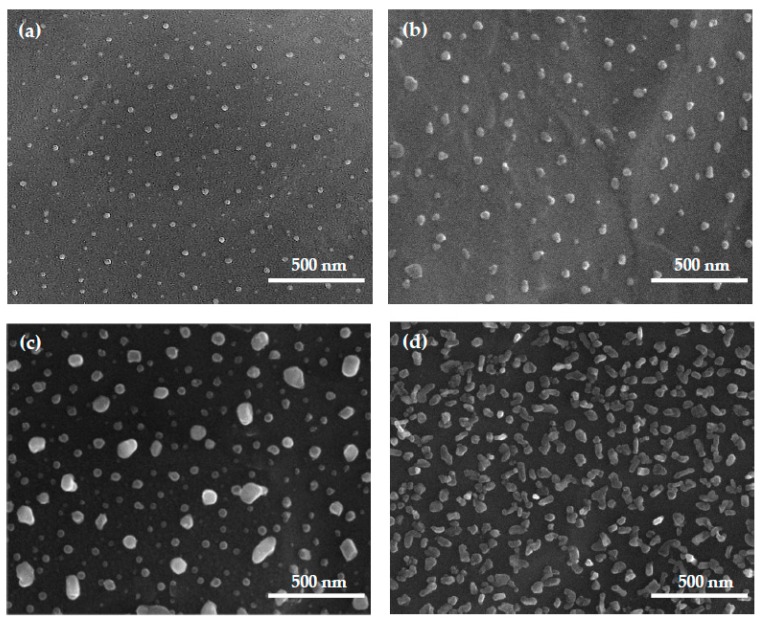
The scanning electron microscopy (SEM) images of Ti6Al4V/0.9AgNPs; *d* = 18 ± 8 nm (**a**), Ti6Al4V/1.1AgNPs; *d* = 45 ± 15 nm (**b**), Ti6Al4V/1.3AgNPs; *d* = 68 ± 32 nm (**c**), Ti6Al4V/2.3AgNPs; *d* = 53 ± 18 nm (**d**).

**Figure 2:**
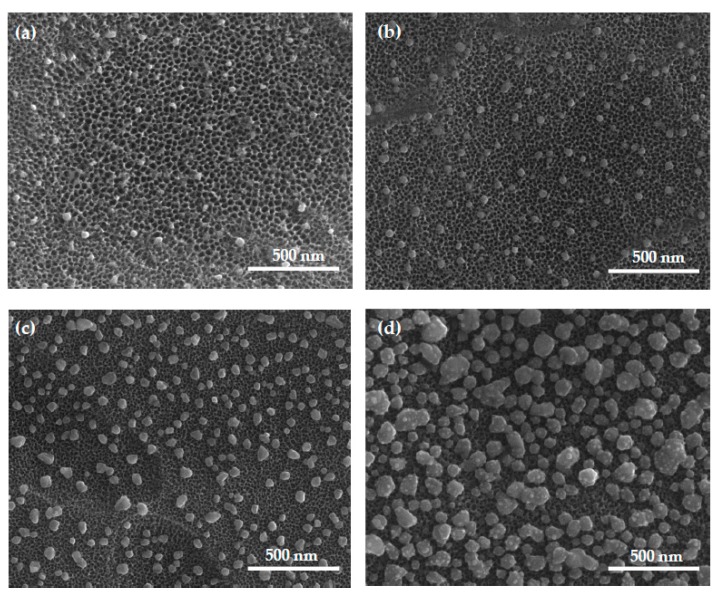
Scanning electron microscopy (SEM) images of Ti6Al4V/TNT5/0.6AgNPs; *d* = 38 ± 14 nm (**a**), Ti6Al4V/TNT5/1.0AgNPs; *d* = 43 ± 10 nm (**b**), Ti6Al4V/TNT5/1.6AgNPs; *d* = 57 ± 24 nm (**c**), Ti6Al4V/TNT5/2.3AgNPs; *d* = 115 ± 49 nm (**d**).

SEM images of the Ti6Al4V/0.9–2.3AgNPs and Ti6Al4V/TNT5/0.6–2.3AgNPs samples are presented in Figure 1 and Figure 2. Analysis of these data shows that the precursor mass applied in the CVD experiments and the substrate type are two main factors directly impacting the size and distribution of the deposited nanoparticles (Table 2).

The analysis of the SEM images of Ti6Al4V substrates (Figure 1a–d), whose surfaces have been enriched with AgNPs, shows that this surface is evenly covered by dispersed silver grains, whose densities increase with the increase of the nanoparticles weight percent on the substrate surface. The diameter of the nanosilver grains ranges from 18 ± 8 nm up to 53 ± 18 nm. The smallest diameter of nanosilver is observed when 5 mg of the precursor was used. The use of 20 mg of the CVD precursor in the same deposition conditions led to the formation of AgNPs with significant differences in the diameter (from 45 up to 90 nm) and shape (from spherical to rods) of silver grains (Figure 1c). These deposition conditions are probably suitable for the nucleation of spherical grains and their later growth in one direction (formation of rods). The increase of the CVD precursor concentration in vapors (50 mg) caused the deposition of AgNPs of similar diameter and shape, but their arrangement is characterized by a significantly higher density (Figure 1d).

SEM images of the Ti6Al4V/TNT5/AgNPs systems are presented in Figure 2a–d. Their analysis shows that on the surface of the TNT5 coating (tubes diameter allow c.a. 28 ± 11 nm), the diameter of the deposited AgNPs changes from 38 ± 14 nm up to 115 ± 49 nm. Both the size of the nanoparticles’ diameters and their density on the surface of nanotubes grow along with the increase of the number of silver precursor used in the deposition process. Compared to the growth of the silver nanoparticles on the unmodified surface of the titanium alloy, the silver nanoparticles’ growth on the nanotubes is more rational, predictable and does not show any anomalies.

**Figure 3:**
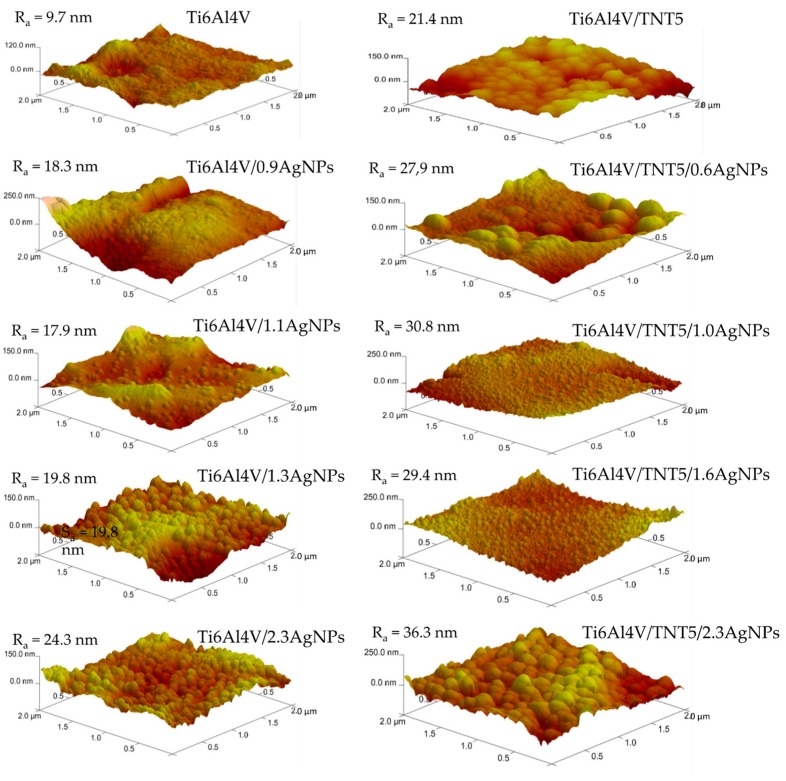
Atomic forces microscopy (AFM) images and R_a_ parameters determined for the Ti6Al4V, Ti6Al4V/AgNPs, Ti6Al4V/TNT5, and Ti6Al4V/TNT5/AgNPs samples.

The surface roughness parameter (R_a_) of the studied samples was measured in the 2 × 2 μm area using software, which is an integral part of the atomic force microscopy (AFM, Veeco (Digital Instruments), Figure 3). The values of the R_a_ parameters determined for Ti6Al4V and Ti6Al4V/TNT5 samples were used as reference samples. The analysis of these data revealed a significant increase of the roughness parameter value with the increase of the density and size of the AgNPs deposited on the surface of both types of substrates. Moreover, the comparison of the Ti6Al4V/TNT5/AgNPs samples and the Ti6Al4V/AgNPs ones indicate the clear influence of the nanotubular architecture on the increase of the surface roughness, e.g., the value of a R_a_ parameter of Ti6Al4V/TNT5/1.0AgNPs is about 42% higher than that of Ti6Al4V/1.1AgNPs. 

**Figure 4:**
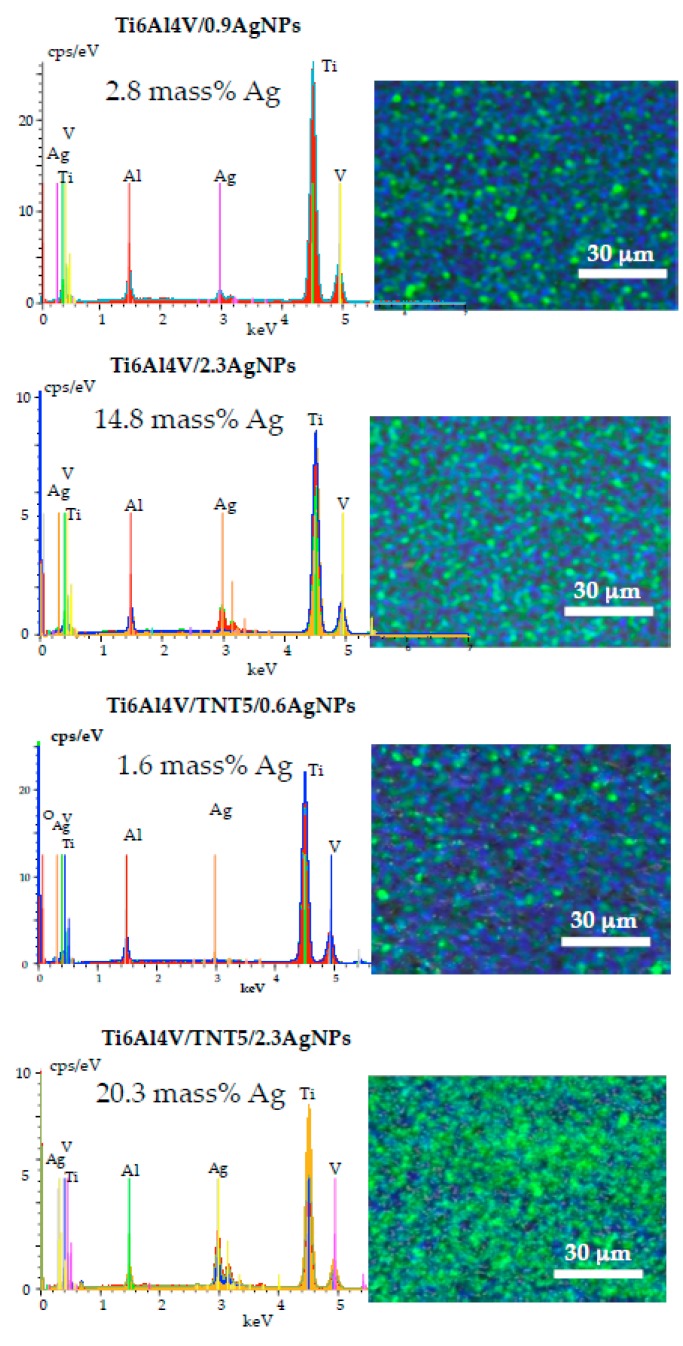
The energy dispersive X-ray spectroscopy (EDS) spectra and maps images of Ti6Al4V/0.9AgNPs, Ti6Al4V/2.3AgNPs, Ti6Al4V/TNT5/0.6AgNPs, and Ti6Al4V/TNT5/2.3AgNPs (AgNPs are marked as the green dots on the presented map images).

In order to confirm the deposition of silver nanograins on the surface of studied substrates, energy dispersive X-ray spectroscopy (EDS) was applied. The low intense lines, which are found in the EDS spectra of Ti6Al4V/0.9AgNPs and Ti6Al4V/TNT5/0.6AgNPs, show the presence of dispersed silver nanoparticles on the surface of the used substrates (Figure 4). The increase of AgNPs’ density on the substrates’ surface and their size caused a significant increase of the integral intensity of the Ag lines in the EDS spectra.

### 3.3. Wettability and Surface Free Energy of Biomaterials

**Figure 5:**
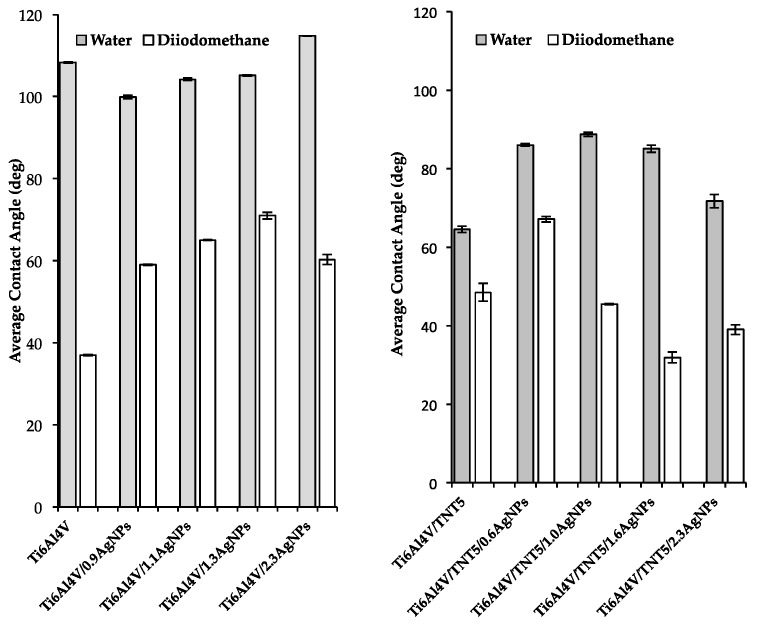
The contact angles values for Ti6Al4V, Ti6Al4V/AgNPs, Ti6Al4V/TNT5, and Ti6Al4V/TNT5/AgNPs.

**Figure 6:**
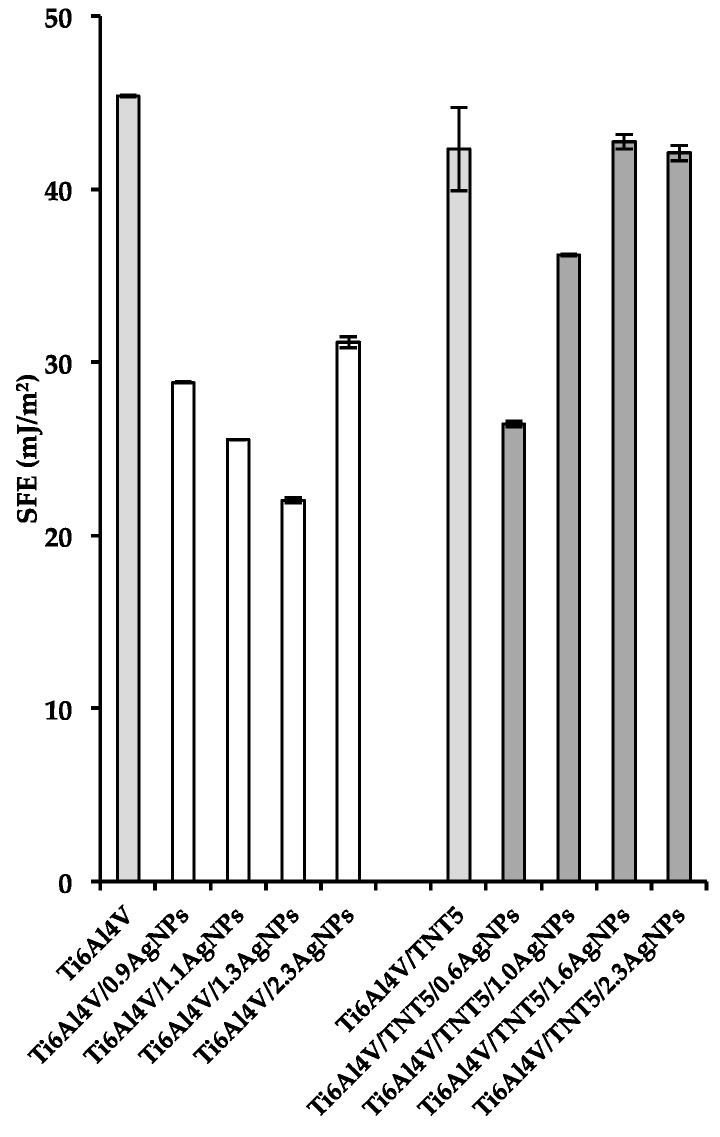
The surface free energy values for Ti6Al4V, Ti6Al4V/AgNPs, Ti6Al4V/TNT5, and Ti6Al4V/TNT5/AgNPs.

In order to estimate the value of the surface free energy based on mathematical calculations, which were performed using the Owens–Wendt method, the contact angle of two different liquids (one of them, polar, and the other one, dispersional) had to be used in the analyses [39]. Therefore, polar water and dispersional diiodomethane were chosen as measuring liquids. The obtained contact angles measurements results, as well as the calculated SFE values, are presented in Figure 5 and Figure 6 and in Table S1. According to these data, the hydrophobic character of the Ti6Al4V surface slightly decreases after depositing small amounts of AgNPs (0.9 wt%), however, with the increase of their density, the coatings’ hydrophobicity increases (Figure 5, Table S1). The fabrication of the titania nanotubes layer on the surface of Ti6Al4V leads to the formation of a hydrophilic surface, which becomes more hydrophobic when it is enriched with silver nanoparticles (Figure 5, Table S1).

The values of the surface free energy (SFE) decreases for all Ti6Al4V/AgNPs samples in comparison to the adequate value for pure Ti6Al4V sample (Figure 6, Table S1). For the sample Ti6Al4V/1.3AgNPs, this is more than two times lower. A different situation is noticed for the Ti6Al4V/TNT and Ti6Al4V/TNT/AgNPs samples. Here, with the exception of the first two silver-enriched systems (Ti6Al4V/TNT/0.6AgNPs and Ti6Al4V/TNT/1.0AgNPs) for which the SFE values are lower than for Ti6Al4V/TNT, two consecutive ones, i.e., Ti6Al4V/TNT/1.6AgNPs and Ti6Al4V/TNT/2.3AgNPs, are characterized by a similar value of free surface energy (Figure 6, Table S1).

### 3.4. Silver Ion Release in the Body Fluid Environment

**Figure 7:**
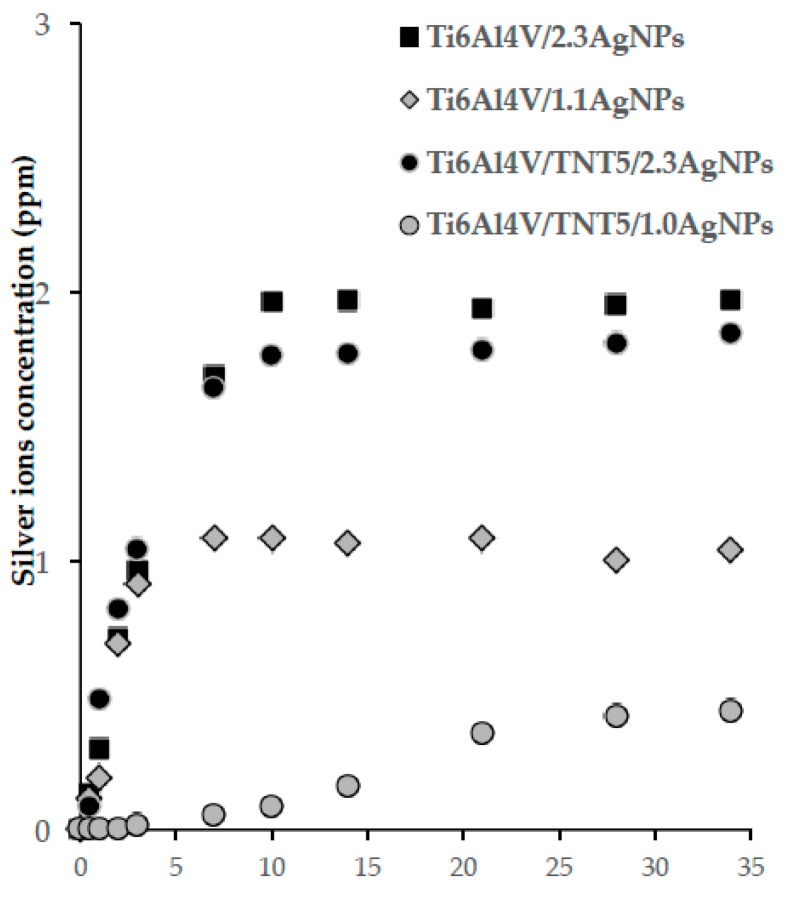
The release amount of Ag^+^ ions from Ti6Al4V/AgNPs and Ti6Al4V/TNT5/AgNPs samples (containing 2.3 and 1.0–1.1 wt% of AgNPs) immersed in a phosphate buffered saline (PBS) and measured by inductively coupled plasma ionization mass spectrometry (ICP-MS).

The bioactivity of Ti6Al4V and Ti6Al4V/TNT5 samples enriched with silver nanoparticles associated with silver ions releasing from their surface can be estimated on the basis of the Ag^+^ ion concentration change studies versus time (5 weeks) for the samples immersed in the phosphate-buffered saline (PBS) solution at human body temperature (37 °C) [36]. Ti6Al4V and Ti6Al4V/TNT5 samples, whose surfaces were enriched with 1.0–1.1 and 2.3 wt% of AgNPs, deposited using 10 and 50 mg of Ag CVD precursor, respectively (Ti6Al4V/1.1AgNPs, Ti6Al4V/2.3AgNPs, Ti6Al4V/TNT5/1.0AgNPs, and Ti6Al4V/TNT5/2.3AgNPs), were used in our studies on the silver ions release effect (Figure 7). The analysis of these data proved the lack of significant differences in the Ag^+^ ion release effect for samples containing a high content of AgNPs on their surface, i.e., Ti6Al4V/2.3AgNPs and Ti6Al4V/TNT5/2.3AgNPs. In both cases, the rapid increase of Ag^+^ concentration in the PBS solution was noticed in the first 10 days of the experiment, and then the concentration changes remained at the level of 1.7–2 ppm. The deposition of nearly a 2-fold smaller amount of AgNPs on the surface of the studied substrates caused a significant reduction in the concentration of silver ions released. In the case of the Ti6Al4V/1.1AgNPs sample, the maximum Ag^+^ release was achieved after 7 days and, in the long-term, it remains at the level of 0.9–1.1 ppm. In the first 10 days, the release of silver ions from the surface of the Ti6Al4V/TNT5/1.0AgNPs sample immersed in the PBS solution was lower than 0.1 ppm. The highest concentration of silver ions was observed after 34 days, i.e., 0.4 ppm. The results of the study showed a significant effect of the number of AgNPs dispersed on the surface of TNT layers on the concentration of silver ions released. 

### 3.5. The Evaluation of the Biocompatibility of the Produced Titanium Alloy-Based Materials

**Figure 8:**
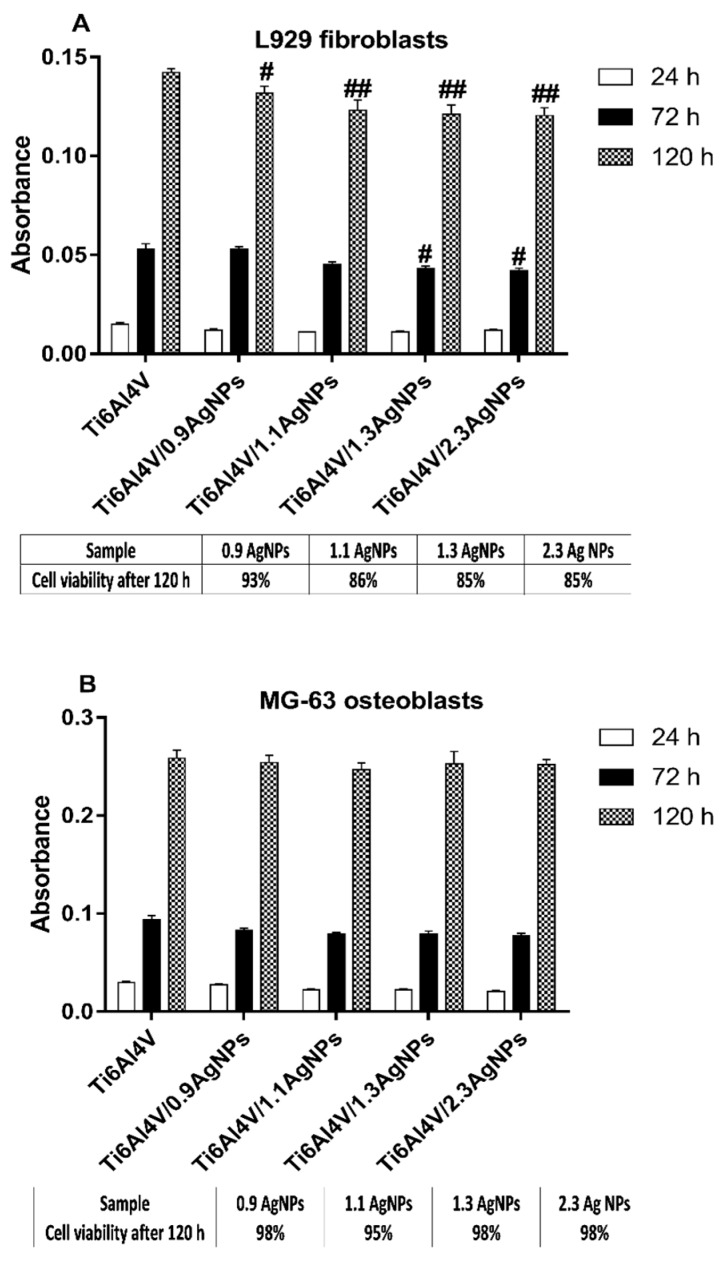
The L929 murine fibroblasts (**A**) and human osteoblasts MG-63 (**B**) adhesion (measured after 24 h) and proliferation (evaluated after 72 h and 120 h) on the surface of Ti6Al4V/AgNPs, detected by the MTT (3-(4,5-dimethylthiazol-2-yl)-2,5-diphenyltetrazolium bromide) assay. The absorbance values are expressed as means ± SEM of five independent experiments. Hash marks indicate significant differences at the appropriate incubation time between the cells incubated on the reference Ti6Al4V alloy foils (Ti6Al4V) compared to the specimen coatings doped by the different concentrations of Ag (# *p* < 0.05, ## *p* < 0.01). Tables below Figure 8A,B presented relative L929 cells or MG-63 cell viability (%) compared to the Ti6Al4V reference sample measured after 120 h of incubation.

**Figure 9:**
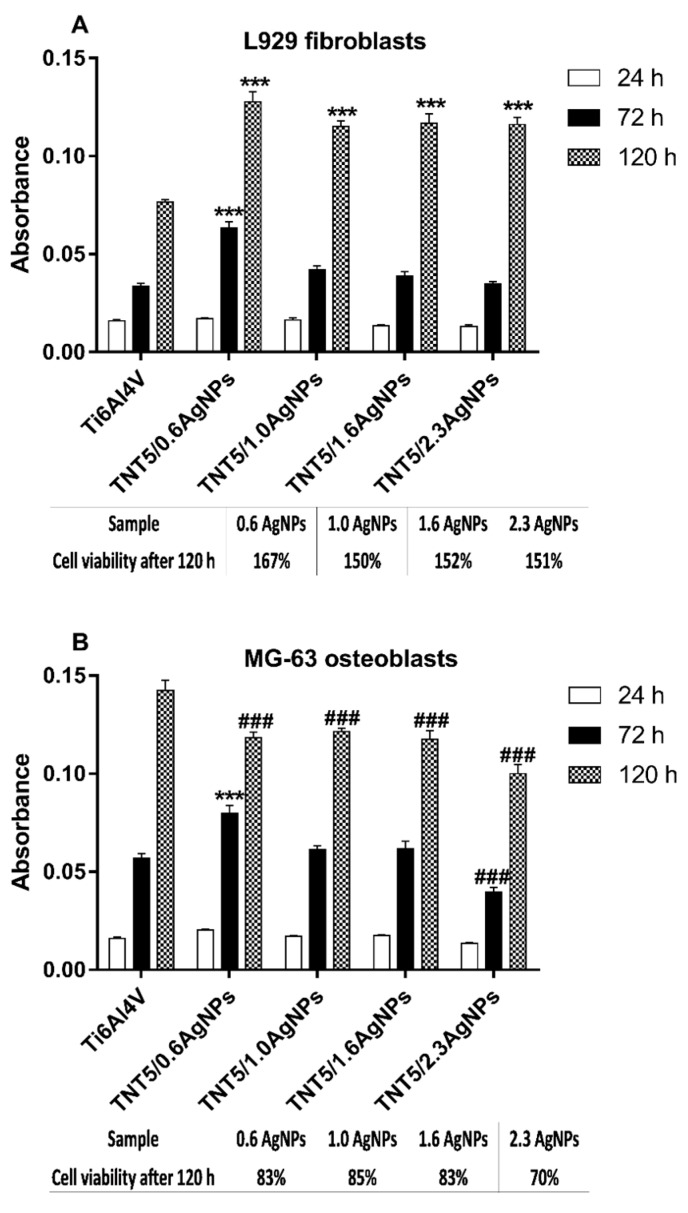
The effect of TNT5/AgNPs coatings on the L929 fibroblasts (**A**) and MG-63 osteoblasts (**B**) adhesion (measured after 24 h) and proliferation (evaluated after 72 h and 120 h), detected by the MTT assay. The absorbance values are expressed as means ± SEM of five independent experiments. Asterisks indicate significant differences at the appropriate incubation time when the level of cell proliferation on the surface of specimens coating doped by the different concentrations of Ag was higher compared to the reference Ti6Al4V alloy foils (Ti6Al4V) (*** *p* < 0.001). Hash marks denote significant differences at the appropriate incubation time when the level of cell proliferation on the samples enriched with AgNPs was lower in comparison with the reference Ti6Al4V alloy foils (### *p* < 0.001). Tables below Figure 9A,B presented relative L929 or MG-63 cells viability (%) compared to the Ti6Al4V reference sample measured after 120 h of incubation.

The biocompatibility of the studied substrates, whose surfaces were enriched with different amounts of dispersed silver nanoparticles, were evaluated on the basis of the MTT (3-(4,5-dimethylthiazol-2-yl)-2,5-diphenyltetrazolium bromide) assay and Scanning Electron Microscopy (SEM) micrographs. The assays were made for Ti6Al4V, Ti6Al4V/AgNPs and Ti6Al4V/TNT5/AgNPs, using two cell lines: the murine fibroblast cell line L929 and human osteoblast-like MG-63 cells. The level of adhesion (measured after 24 h) and proliferation (assessed after 72 h and 120 h) of the cells growing on the surface of Ti6Al4V/AgNPs and Ti6Al4V/TNT5/AgNPs was compared to that which was observed for the cells cultured on the reference Ti6Al4V alloy foil. As it can be seen, with an increase of the incubation time, more L929 fibroblasts, as well as MG-63 osteoblasts, proliferated on the surface of all the tested biomaterials (Figure 8 and Figure 9). Analysis of the MTT assay data revealed the lack of significant differences in the MG-63 osteoblasts adhesion and proliferation to the surface of the reference Ti6Al4V sample and Ti6Al4V/AgNPs samples, whose surfaces were enriched with various amounts of AgNPs (Figure 8). In contrast, we have noticed that the Ti6Al4V alloy foils covered with nanosilver provoked a slight decrease in the L929 fibroblasts’ proliferation after 72 h of incubation ((for Ti6Al4V/1.3AgNPs and Ti6Al4V/2.3AgNPs) Ag nanolayers; *p* < 0.05) and 120 h of incubation for the all tested concentration of nanosilver (relative L929 cells’ viability compared to the Ti6Al4V reference sample and expressed as a percentage was presented in the Table below Figure 8A). However, we did not observe any differences in the fibroblasts adhesion measured after 24 h of incubation (Figure 8A). 

The results of the MTT assay for Ti6AlV/TNT5/AgNPs are presented in Figure 9. The results were compared to that which was observed for the cells cultured on the reference Ti6Al4V alloy foils. Analysis of these data showed no differences in the cell adhesion (measured after 24 h) for both the tested cells lines. Moreover, L929 fibroblasts cultured on the surface of TNT5 nanotubes’ coatings doped by the all tested concentrations of nanosilver showed a higher rate of cell proliferation after 120 h of incubation than the cells that grew on the Ti6Al4V reference layers (*p* < 0.001).

This phenomenon was also observed after 72 h of incubation, but only for the samples enriched with 0.6 wt% of AgNPs (*p* < 0.001, Ti6Al4V/TNT5/0.6AgNPs). On the other hand, MG-63 osteoblasts cultured on the TNT5 nanotubes enriched with silver nanoparticles provoked a decrease in the level proliferation of MG-63 osteoblasts after 120 h of incubation (*p* < 0.001) in comparison to the Ti6Al4V reference alloy (*p* < 0.001) (the relative MG-63 cells viability compared to the Ti6Al4V reference sample and expressed as a percentage was presented in the Table below the Figure 9B). However, it should be clearly emphasized that these samples also showed an increase in the level of proliferation over time.

**Figure 10:**
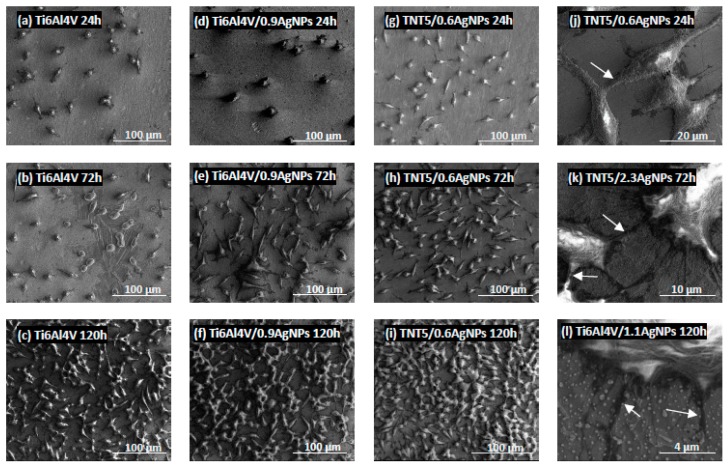
The scanning electron microscopy (SEM) images presenting adhesion (after 24 h) and proliferation (after 72 h and 120 h) of the murine L929 fibroblasts growing on the surface of reference Ti6Al4V alloy foils (**a**–**c**), (Ti6Al4V/0.9AgNPs) (**d**–**f**) or Ti6Al4V/TNT5/0.6AgNPs (**g**–**i**). Arrows in the micrographs indicate numerous filopodia spreading between the fibroblasts (**j**–**k**) or filopodia, which attached the cells to the surface of nanocoatings (**l**).

**Figure 11:**
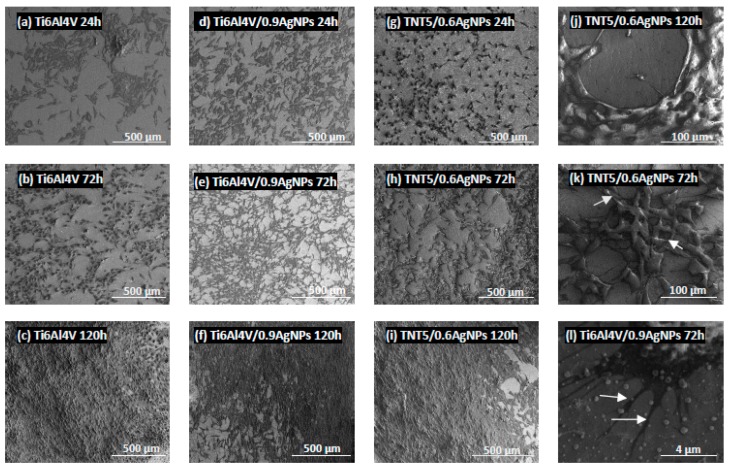
The scanning electron microscopy (SEM) micrographs showing the human osteoblast-like MG-63 cells adhesion (after 24 h) and proliferation (after 72 h and 120 h) growing on the surface of references Ti6Al4V alloy foils (**a**–**c**), (Ti6Al4V/0.9AgNPs) (**d**–**f**) or Ti6Al4V/TNT5/0.6AgNPs (**g**–**i**). The micrograph (**j**) presents the multilayer growth of cells on the surface of Ti6Al4V/TNT5/0.6AgNPs sample. Arrows indicate numerous filopodia, which attached the osteoblasts to the nanocoatings surface (**l**) and filopodia spreading between the cells (**k**).

Biointegration of titania nanolayers enriched with silver nanoparticles were also assessed by the analysis of SEM micrographs. Comparative SEM images showing the morphology, adhesion and proliferation of the cells growing on the surface of Ti6Al4V alloy and Ti6Al4V/AgNPs, as well as on Ti6Al4V/TNT5/AgNPs, are presented in Figure 10 and Figure 11, respectively. These data clearly demonstrate the high biocompatibility of both types of tested nanolayers, supporting the results from the MTT assay. As it can be seen, L929 fibroblasts cultured on the surface of Ti6Al4V/AgNPs, as well as on the surface of Ti6Al4V/TNT5/AgNPs, showed the increase in cell proliferation over time (compare micrographs in Figure 10d–i). A similar phenomenon was also observed for the MG-63 osteoblasts (compare micrographs in Figure 11d–i). Importantly, the cells, especially MG-63 osteoblasts, start to grow in layers on top of each other (Figure 11k), and moreover, the cells grow with a multilayer structure on most of the surfaces of the nanolayers after 120 h of incubation time (see micrographs in Figure 11f,i,j). Finally, the SEM images also show that L929 fibroblasts, as well as MG-63 osteoblasts, form numerous filopodia which attach the cells to the surface of specimens by penetrating deep into the nanolayers (arrows in Figure 10l and Figure 11l, respectively). These thin, actin-rich plasma-membrane protrusions were also generated between the cells (arrows in Figure 10j–k and Figure 11k, respectively).

### 3.6. Antimicrobial Activity of Silver-Coated Titanium Alloys

The antimicrobial activity of silver nanoparticles is widely known. Titanium alloys (surface non-modified and nanotubular modified), enriched with various amount of nanosilver grains (Ti6Al4V/AgNPs and Ti6Al4V/TNT5/AgNPs) were found to be extremely biocidal against the tested bacteria and fungi when compared to silver non-coated titanium alloy. Biocidal activity was found after 24 h, 14 days and 28 days of silver ion release into PBS. The Ti6Al4V/AgNPs and Ti6Al4V/TNT5/AgNPs composites reduced more than 99% of the growth of all tested microorganisms, independently from the number of silver nanoparticles deposited on their surface (Table 3, Table 4 and Table 5). The number of bacterial colonies after treatment with Ti6Al4V/AgNPs and Ti6Al4V/TNT5/AgNPs was reduced at least 100-fold when compared to Ti6Al4V, as presented in Figure S1 for *E. coli* ATCC25922 7.0 × 10^5^ c.f.u. mL^−1^ (**a**) and 3.8 × 10^5^ c.f.u. mL^−1^ (**b**), respectively.

## 4. Discussion

The studies on the relationship between the number of silver nanoparticles (AgNPs) deposited on the surface of Ti6Al4V and Ti6Al4V/TNT5 substrates, their size and their distribution, and the wettability and bioactivity of the produced systems were the purpose of our investigations. The following two factors determined the choice of the substrate used in our research: (a) the common use of the Ti6Al4V alloy as a material in implantology, and (b) the use of titania nanotube coatings (TNT) to modify the titanium/titanium alloys surfaces and to provide them with biocompatible properties. The electrochemical anodization method, with the use of constant potential (U = 5 V), was applied in the TNT5 coatings production. The results of our earlier works revealed that the TNT5 layer consists of densely packed titania nanotubes of diameters 35–45 nm and length c.a. 150 nm. Simultaneously, this type of coating exhibited optimal biointegration properties [34]. The above-mentioned coating enrichment with AgNPs using the CVD technique lad to the deposition of the dispersed metallic grains mainly on their surface, as in the case of the pure alloys substrates [36]. To achieve better control over the dispersion and growth of deposited AgNPs, low flow values of precursor vapors over the substrate surface were used during the CVD process. Depending on the precursor mass applied in the CVD experiments (i.e., 5, 10, 20, 50 mg) and the defined carrier gas flow, the amounts of the precursor which flow above the substrate surface, were 0.2, 0.3, 0.7, and 1.7 mg·min^−1^, respectively. The analysis of the SEM images confirmed the clear influence of the experimental conditions on the size and density of the deposited AgNPs (Table 2 and Figure 1 and Figure 2). Moreover, it should be noted that the type of used substrate also affects the increase of the deposited AgNPs’ size and density. The diameter of the AgNPs deposited on the surface of Ti6Al4V/TNT5 was bigger than the ones, which were deposited on the surface of the Ti6Al4V substrates (Table 2).

In our work, the biointegration of the studied samples was evaluated using two cell lines: mouse L929 fibroblasts and human osteoblasts-like MG-63 cells. According to earlier reports, the long-term success of an implant placement depends not only on the integrity of osseointegration, but also on the contact with the surrounding soft tissue [40]. In recent years, the MG-63 cell line has become a standard model for bone research in addition to primary human osteoblasts and this cell line is also well-established for studying the effects of surface nanotopography on osteoblast-like cells [41]. In addition, established permanent cell lines of soft tissue, such as L929 fibroblasts, are widely used to test the cytotoxicity of dental materials when employing in vitro methods of experimentation [42]. Moreover, fibroblasts are the most common cells in connective tissue, one of the main components of peri-implant soft tissue, which is key to the formation of the peri-implant mucosal seal and helping to prevent epithelial ingrowth [40]. Therefore, the study of the biointegration of the nanomaterials using two selected cell lines allowed for a comprehensive examination of implant biocompatibility.

It is well-established that cellular behavior, such as cell adhesion and proliferation or morphologic change (including the formation of filopodia) is determined by the surface properties of nanomaterials, thus, the cellular response measured by these parameters are required to assess the biointegration of implants [43]. In our study, the biointegration level of the tested specimens was examined using an MTT assay (for evaluation of cell adhesion and proliferation) and scanning electron microscopy images analysis (for assessment of cell adhesion, proliferation and morphology). The results of the MTT assays related to the cell adhesion (measure after 24 h) and proliferation (measured after 72 h and 120 h) revealed that there were no differences in the MG-63 osteoblasts adhesion and proliferation between the reference Ti6Al4V layers and Ti6Al4V/AgNPs (Figure 8B). On the other hand, the Ti6Al4V/AgNPs induced a significant decrease in the L929 fibroblast proliferation, especially after 120 h of incubation for the all tested concentration of nanosilver (Figure 8A). Surprisingly, the different results were obtained for Ti6Al4V/TNT5/AgNPs samples. L929 fibroblasts cultured on the surface of Ti6Al4V/TNT5 samples enriched with all tested concentration of AgNPs showed a higher rate of cell proliferation after 120 h of incubation than the cells that grow on the Ti6Al4V reference layers (Figure 9A). In contrast, the same nanolayers induced a decrease in the level of proliferation of the MG-63 osteoblasts after 120 h of incubation (Figure 9B). However, it should be clearly emphasized that for the all tested samples, we have noticed an increase in the L929 fibroblast and MG-63 osteoblast proliferation over time, which is confirmed not only by the MTT assay results, but also through the analysis of the comparative SEM micrographs (compare the images in Figure 10 and Figure 11). Importantly, the cells, especially the MG-63 osteoblasts, have almost overgrown the entire surface of the nanolayers enriched with AgNPs (Figure 11f,i,j). The high level of biocompatibility of the tested nanomaterials was also confirmed by the cellular behavior associated with the formation of filopodia by the fibroblasts, as well as the osteoblasts, between the cells (arrows in Figure 10j,k and Figure 11k, respectively). These actin-based cell protrusions also attached the cells to the coating’s surface (arrows in Figure 10l and Figure 11l, respectively) by penetration inside the porous nanolayer, functioning as anchorage points enhancing cell proliferation. Filopodia are regarded as one of the most important cellular sensors, collecting information on whether the surface is suitable for cell attachment and proliferation, cell-cell interacting and allowing for cell migration toward the destination [44]. Therefore, filopodia formation is evidence of the biocompatible properties of tested nanomaterials. 

Although it is believed that silver has cytotoxicity to some cells at certain concentrations [45], it is well-known that eukaryotic cells exhibit a far bigger target for attacking silver ions than prokaryotic cells and that they show more structural and functional redundancy. Therefore, a higher silver ion concentration is required to achieve comparable toxic effects, relative to bacterial cells [46]. Similar to our results, Reference [47] demonstrated that Ag-decorated TiO_2_ nanotubes exhibited monotonically increasing trend in the fibroblasts’ cell line proliferation and, at the same time, these specimens may cause a decrease in osteoblast proliferation [48]. Importantly, in our study, the viability of MG-63 osteoblasts cultured on the TNT5 samples enriched with nanosilver was 70% or more after 120 h of incubation in comparison to the Ti6Al4V references alloy (Table below Figure 9B). According to the ISO 10993-1:2018 standards [49] (Biological evaluation of medical devices: Part 1: evaluation and testing within a risk management process), if the cell viability was reduced to <70% of the blank, it would have a cytotoxicity potential. Therefore, our results indicate reasonable biocompatibility of TNT5 enriched with all tested concentration of nanosilver. As we have described above, Ti6Al4V alloy foils enriched with all tested concentrations of silver nanograins induced a slight decrease in L929 fibroblast proliferation without affecting the proliferation of osteoblasts. However, these results also demonstrate reasonable biocompatibility of the tested nanomaterials because the viability of the cells after 120 h of incubation was 85% or more compared to the reference Ti6Al4V specimens (Table below the Figure 8A). Our results corresponding with the findings from the other authors who have shown that silver deposited titanium reduced fibroblasts proliferation by 20% in comparison with titanium control samples [50] or titanium samples coated with the silver alloys, which did not have a cytotoxic effect on osteoblast cells [51]. 

To summarize, our results from the MTT assay and analysis of comparative SEM micrographs, including an increase in the cell proliferation over time, filopodia formation and viability of cell higher than 70% for the all tested samples, clearly demonstrate the biocompatible properties of the tested nanomaterials that can be used in dentistry or maxillofacial surgery. This conclusion is based on the statement that a favorable cellular interaction with the biomaterial’s surface is critical for the long-term success of the implants [52].

Implant-associated infections are one of the critical issues for dental and maxillofacial implantology and can result in serious complications, such as the need for complex revision procedures, as well as poor prognoses, patients suffering, and even death [53,54]. The infections associated with implants are caused mainly by bacterial colonization and biofilm formation on the surface of the implanted specimen, which affects the adjacent tissues [55]. It is known that the most effective way to prevent biofilm buildup on implants is to prohibit the initial bacterial adhesion because the biofilms are quite difficult to remove after formation. One of the approaches is to directly impregnate an implant device with antibiotics to prevent the initial adhesion of bacteria onto the implant surface [53,56]. Although these antibiotic-impregnated surfaces displayed significant therapeutic effects, the potential toxicity and increased microbial drug resistance through the slow-release doses have become increased risks in surgery [53,55]. Therefore, postoperative infection rates could be greatly reduced by improving the antimicrobial properties of the implant surface by its modifications with metal ions such as Ag and Zn [54,55,57]. Silver-containing coatings have attracted increasing attention due to the nontoxicity of the active Ag^+^ to human cells and its antimicrobial activity [53,58]. Thus, the surface-modification of titanium alloys with silver coating, which was also performed in the present study, is considered a strategy to prevent the development of peri-implant infections [54]. Based on the results obtained from ICP-MS, which showed a significant release of silver ions from all Ti6Al4V/AgNPs and Ti6Al4V/TNT5/AgNPs immersed into a PBS solution after 14 and 28 days, we presumed that such ions could be responsible for antimicrobial properties. Although superior microbiocidal activity was also observed for all the studied samples after a 24-h ion release time, the ICP-MS did not confirm the presence of silver ions in the case of nanotubular modified titanium alloy surfaces enriched with the smallest amount of silver (Figure 5). This might be due to the low content of released ions, which was not detectable. Thus, the limited sensitivity of ICP-MS is not without meaning. A high biocidal effect of Ti6Al4V/AgNPs and Ti6Al4V/TNT5/AgNPs was observed even at the lowest concentrations of Ag deposited on the surface of the tested alloys. We assert that the required Ag dose in the implants is typically low, which makes it possible to introduce Ag into biocompatible coatings [55]. Therefore, the incorporation of a sufficient amount of Ag to enhance the antibacterial ability of porous coatings could lead to the production of a surface that retains biocompatible and relatively long-term antibacterial activity. On the other hand, the optimization of the fabrication of Ti-Ag specimens by a decrease of the Ag amount on their surface might also improve the adhesion and proliferation of fibroblasts and osteoblasts, thus affecting the better integration of implants with human tissues. 

We assert that the inhibitory effect of silver nanoparticles is mainly associated with silver ions present in nanoparticles, but it is not the sole mechanism of antimicrobial activity induced by nanosilver [59]. The major difference between the effectiveness of silver nanoparticles and silver ions against bacteria is that AgNPs act in nanomolar concentrations, while ions act in micromolar ranges [60]. Silver nanoparticles, due to their small size, can easily penetrate and disrupt the membranes of bacteria. Both silver species (nanoparticles and ions) may react with protein thiol groups (key respiratory enzymes) and/or phospholipids of the bacterial membrane [61,62,63]. This leads to an increase in the membrane permeability and may cause more pronounced effects such as the loss (by leakage) of cellular contents, including ions (mainly K^+^), proteins and reducing sugars and a decrease of the ATP level [60,64,65]. Silver species may also interact with nucleic acids, which may probably result in the impairment of DNA replication [59,60,66,67]. All of these effects may culminate in the loss of cell viability [60,68]. It is also suggested that silver ions generate free radicals inside cells, which are involved in the antimicrobial activity of silver nanoparticles and released silver ions [69]. Some authors claimed that the thickness of the peptidoglycan layer of gram-positive bacteria might prevent the action of the silver ions as they found a higher inhibitory activity from the silver ion solution against *E. coli* than against *S. aureus* [70]. However, the microbiocidal activity of silver nanoparticles has been found against both Gram-positive (e.g., *Staphylococcus aureus*) or Gram-negative (e.g., *Escherichia coli*) and even yeasts [69,71,72], which is consistent with our results. The results of our study, where surface modified titanium alloys affected the inhibition of the growth of both Gram-positive and Gram-negative bacteria or fungi, are in good accordance with a previous report [58] where TiO_2_ nanotubes enriched with Ag demonstrated superior bactericidal properties against the planktonic bacteria. Similar findings were reported by Reference [54]. The authors showed the strong bactericidal activity that titanium specimens incorporated with silver against *Staphylococcus aureus*. Moreover, the number of bacteria decreased as the dosage of the incorporated Ag increased, suggesting that the antibacterial ability increased with the content of deposited Ag [54].

## 5. Conclusions

The combination of antibacterial ability and biocompatibility, as well as non-cytotoxicity, studied in vitro, indicates that the optimal AgNPs enriching method could provide a promising strategy for the fabrication of a long-term antibacterial surface and, thus, an attractive biomaterial which successfully solves the growing problem of peri-implant infection. By taking into account the obtained results, it can be stated that all studied samples revealed very high antimicrobial activity, resulting from the release of Ag^−^ ions from silver nanoparticles, as well as high biocompatibility. Moreover, they are all characterized by a relatively simple synthesis. However, among studied systems, Ti6Al4V/TNT5/0.6AgNPs contained the lowest amount of AgNPs, but revealed to still have optimal biointegration properties and high biocidal properties. Thus, it can be taken into account as a biomaterial possessing the desired biological properties and, at the same time, as a biomaterial in which the potential harm is minimized by minimizing the number of silver nanoparticles. 

## Figures and Tables

**Table 1:** The silver nanograins CVD parameters.

Precursor	Ag_5_(O_2_CC_2_F_5_)_5_(H_2_O)_3_
Precursor weight	5, 10, 20, 50
Vaporization temperature (T_V_)	230
Carrier gas	Ar
Total reactor pressure (p)	3.0
Flow of the precursor vapors above the substrate	0.2–1.7
Substrate temperature (T_D_)	290
Substrates	Ti6Al4V and Ti6Al4V/TNT5
Deposition time	30

CVD: chemical vapor deposition.

**Table 2:** The weight % and the diameters of the AgNPs deposited on the surface of the Ti6Al4V and Ti6Al4V/TNT5 substrates using the CVD technique.

	Ti6Al4V/AgNPs		Ti6Al4V/TNT5/AgNPs	
Precursor Mass (mg)	wt%	*d* (nm)	wt%	*d* (nm)
**5**	0.9	18 ± 8	0.6	38 ± 14
**10**	1.1	45 ± 15	1.0	43 ± 10
**20**	1.3	68 ± 32	1.6	57 ± 24
**50**	2.3	53 ± 18	2.3	115 ± 49

**Table 3:** The reduction of microbial growth (%) in PBS after the use of Ti6Al4V/AgNPs and Ti6Al4V/TNT5/AgNPs alloys for 24 h of ion release.

	Microorganisms				
Ti Alloys	*E. coli* ATCC8739	*E. coli* ATCC25922	*S. aureus* ATCC6538	*S. aureus* ATCC25923	*C. albicans* ATCC10231
Ti6Al4V	-	-	-	-	-
Ti6Al4V/0.9 AgNPs	99.57	>99.99	>99.99	99.93	99.96
Ti6Al4V/1.1 AgNPs	99.97	99.99	>99.99	99.98	99.97
Ti6Al4V/1.3 AgNPs	99.94	99.80	99.93	99.96	99.67
Ti6Al4V/2.3 AgNPs	99.96	99.83	99.99	99.85	99.93
Ti6Al4V/TNT5	-	-	-	-	-
Ti6Al4V/TNT5/0.6 AgNPs	99.90	99.94	99.94	99.99	>99.99
Ti6Al4V/TNT5/1.0 AgNPs	>99.99	>99.99	99.61	>99.99	>99.99
Ti6Al4V/TNT5/1.6 AgNPs	99.95	99.90	99.46	>99.99	99.99
Ti6Al4V/TNT5/2.3 AgNPs	99.70	99.99	99.95	99.71	>99.99

Key: -; no reduction (control). PBS: phosphate buffered saline solution.

**Table 4:** The reduction of microbial growth (%) in PBS after the use of Ti6Al4V/AgNPs and Ti6Al4V/TNT5/AgNPs alloys for 14 days of ion release.

	Microorganisms				
Ti Alloys	*E. coli* ATCC8739	*E. coli* ATCC25922	*S. aureus* ATCC6538	*S. aureus* ATCC25923	*C. albicans* ATCC10231
Ti6Al4V	-	-	-	-	-
Ti6Al4V/0.9 AgNPs	>99.99	>99.99	>99.99	>99.99	>99.99
Ti6Al4V/1.1 AgNPs	>99.99	>99.99	>99.99	>99.99	>99.99
Ti6Al4V/1.3 AgNPs	>99.99	>99.99	>99.99	>99.99	>99.99
Ti6Al4V/2.3 AgNPs	>99.99	>99.99	>99.99	>99.99	>99.99
Ti6Al4V/TNT5	-	-	-	-	-
Ti6Al4V/TNT5/0.6 AgNPs	>99.99	>99.99	>99.99	>99.99	>99.99
Ti6Al4V/TNT5/1.0 AgNPs	>99.99	>99.99	>99.99	>99.99	>99.99
Ti6Al4V/TNT5/1.6 AgNPs	>99.99	>99.99	>99.99	>99.99	>99.99
Ti6Al4V/TNT5/2.3 AgNPs	>99.99	>99.99	>99.99	>99.99	>99.99

Key: -; no reduction (control).

**Table 5:** The reduction of microbial growth (%) in PBS after the use of Ti6Al4V/AgNPs and Ti6Al4V/TNT5/AgNPs alloys for 28 days of ion release.

	Microorganisms				
Ti Alloys	*E. coli* ATCC8739	*E. coli* ATCC25922	*S. aureus* ATCC6538	*S. aureus* ATCC25923	*C. albicans* ATCC10231
Ti6Al4V	-	-	-	-	-
Ti6Al4V/0.9 AgNPs	99.94	>99.99	99.78	99.58	>99.99
Ti6Al4V/1.1 AgNPs	99.84	99.98	99.91	99.49	>99.99
Ti6Al4V/1.3 AgNPs	99.91	99.98	99.84	99.87	>99.67
Ti6Al4V/2.3 AgNPs	99.89	99.83	99.83	99.87	>99.99
Ti6Al4V/TNT5	-	-	-	-	-
Ti6Al4V/TNT5/0.6 AgNPs	>99.99	>99.99	>99.99	>99.99	>99.99
Ti6Al4V/TNT5/1.0 AgNPs	>99.99	99.99	99.99	99.99	>99.99
Ti6Al4V/TNT5/1.6 AgNPs	99.90	99.84	99.99	>99.99	>99.99
Ti6Al4V/TNT5/2.3 AgNPs	99.66	99.88	99.81	99.76	>99.99

Key: -; no reduction (control).

## Supplementary Materials

The following are available online at https://www.mdpi.com/2077-0383/8/3/334/s1, Table S1: The results of the contact angle measurements, which were made three times using distilled water and diiodomethane and the results of the surface free energy (SFE) of biomaterials used in Owens-Wendt method, Figure S1: Reduction of colony number of *Escherichia coli* ATCC25922=PCM2057 after treatment with silver ions released from Ti6Al4V/AgNPs (b–e) and Ti6Al4V/TNT5/AgNPs (g–j) for 24 h. Number of bacterial colonies after treatment with Ti6Al4V/AgNPs and Ti6Al4V/TNT5/AgNPs was reduced at least 100 fold when compared to Ti6Al4V (7.0 × 10^5^ c.f.u. mL^−1^) (a) and Ti6Al4V/TNT5 (3.8 × 10^5^ c.f.u. mL^−1^) (b).
