# Supplementary material for: “To Be Microbiocidal and Not to Be Cytotoxic at the Same Time…”—Silver Nanoparticles and Their Main Role on the Surface of Titanium Alloy Implants
Source: J Clin Med. 2019 Mar 10;8(3):334. doi: 10.3390/jcm8030334 (PMC6463051; doi:10.3390/jcm8030334)
Supplement: Supplementary file 1 [file jcm-08-00334-s001.pdf]

Table S1. The results of the contact angle measurements, which were made three times using distilled water and diiodomethane and the results of the surface free energy (SFE) of biomaterials used in Owens-Wendt method.

Biomaterial Sample	Average Contact Angle [°] ± Standard Deviation		SFE [mJ/m²]
	Measuring Liquid		
	Water	Diiodomethane	
Ti6Al4V	108.3 ± 0.09	37 ± 0.16	45.37 ± 0.05
Ti6Al4V/0.9AgNPs	99.9 ± 0.44	59.0 ± 0.11	28.83 ± 0.04
Ti6Al4V/1.1AgNPs	104.2 ± 0.32	65.0 ± 0.12	25.53 ± 0.03
Ti6Al4V/1.3AgNPs	105.2 ± 0.13	71.0 ± 0.83	22.02 ± 0.17
Ti6Al4V/2.3AgNPs	114.8 ± 0.06	60.3 ± 1.24	31.14 ± 0.30
Ti6Al4V/TNT5	64.5 ± 0.8	48.5 ± 2.3	42.3 ± 2.04
Ti6Al4V/TNT5/0.6AgNPs	86.0 ± 0.34	67.1 ± 0.68	26.43 ± 0.17
Ti6Al4V/TNT5/1.0AgNPs	88.7 ± 0.54	45.5 ± 0.15	36.18 ± 0.06
Ti6Al4V/TNT5/1.6AgNPs	85.1 ± 0.93	31.9 ± 1.40	42.73 ± 0.43
Ti6Al4V/TNT5/2.3AgNPs	71.7 ± 1.69	39.0 ± 1.25	42.09 ± 0.46

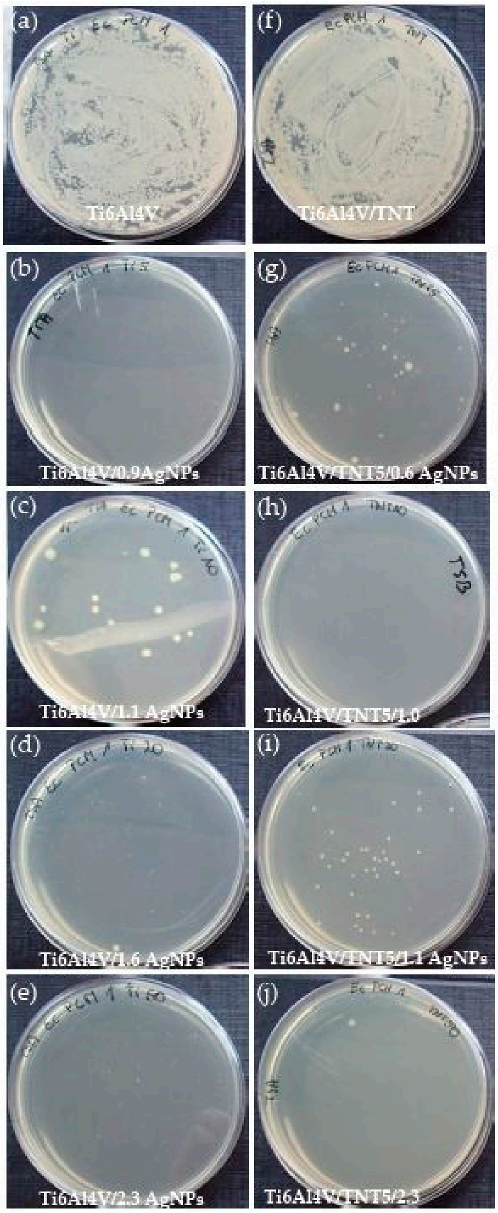

Figure S1. Reduction of colony number of *Escherichia coli* ATCC25922=PCM2057 after treatment with silver ions released from Ti6Al4V/AgNPs (**b–e**) and Ti6Al4V/TNT5/AgNPs (**g–j**) for 24 h. Number of bacterial colonies after treatment with Ti6Al4V/AgNPs and Ti6Al4V/TNT5/AgNPs was reduced at least 100 fold when compared to Ti6Al4V (7.0 x 10^5 c.f.u. ml^{-1}) (**a**) and Ti6Al4V/TNT5 (3.8 x 10^5 c.f.u. ml^{-1}) (**b**).
